# Massive Gouty Tophi Presenting as Pseudotumor of the Elbow: A Rare Presentation

**DOI:** 10.7759/cureus.6769

**Published:** 2020-01-25

**Authors:** Divesh Jalan, Deepak Kumar Maley, Abhay Elhence, Poonam Elhence, Princi Jain

**Affiliations:** 1 Central Institute of Orthopaedics, Vardhman Mahavir Medical College and Safdarjung Hospital, New Delhi, IND; 2 Orthopaedics, MediCiti Institute of Medical Sciences, Hyderabad, IND; 3 Orthopaedics, All India Institute of Medical Sciences, Jodhpur, IND; 4 Pathology, All India Institute of Medical Sciences, Jodhpur, IND; 5 Internal Medicine, Atal Bihari Vajpayee Institute of Medical Sciences and Ram Manohar Lohia Hospital, New Delhi, IND

**Keywords:** gout, tophi, pseudotumor, arthritis

## Abstract

Gout is a systemic metabolic disorder characterized by hyperuricemia and deposition of monosodium urate crystals in joints and other extra-articular tissues. Poorly controlled cases progress to chronic gout with tophi, which can sometimes assume massive sizes. We report one such case of a 39-year-old male with poorly controlled polyarticular tophaceous gout presenting with a massive swelling of the left elbow simulating a soft tissue tumor. Subsequent investigations confirmed it to be a massive tophus which was then surgically excised, as the mass was not responding to the medical management. At the latest follow-up after two years, the patient has full function of the elbow and gout is well controlled with medications.

## Introduction

Gout is a systemic metabolic disorder with elevated serum urate levels and deposition of monosodium urate crystals in synovial and non-articular tissues resulting in repeated attacks of arthritis [1]. The cutaneous manifestations of gout are represented by intradermal lesions or subcutaneous nodules called tophi, commonly seen in avascular tissue over the ears, olecranon and prepatellar bursae or in acral sites, often associated with tendons. Although tophi are seen in 10% of the patients with chronic gout, literature is sporadic on massive gouty tophi [2,3]. We are presenting a case of massive elbow tophi simulating a tumor in a known patient of chronic gouty arthritis.

## Case presentation

A 39-year-old male patient, a known case of chronic gouty arthritis, presented to the orthopaedic clinic with massive swelling over the extensor aspect of left elbow, measuring 18x10 cm. which was progressively increasing over the past 10 years.The swelling was insidious in onset, slowly progressive with a waxing and waning course with occasional pain. There was no associated fever or any other constitutional symptoms. Past and family history were not significant.

**Figure 1:**
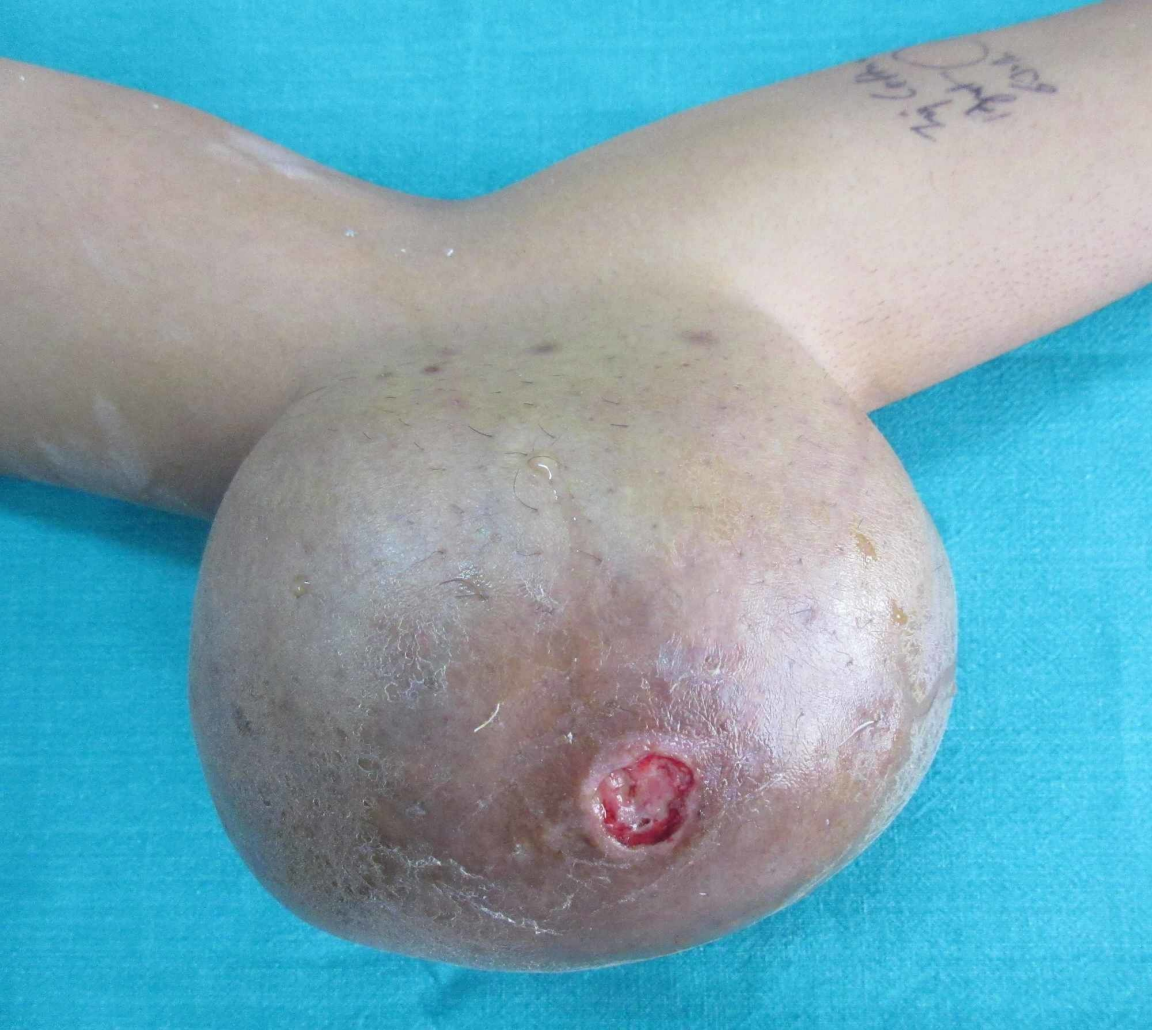
Clinical photograph of the left elbow showing massive swelling with central ulceration.

On physical examination, the skin over the swelling was tense, shiny with venous prominence and superficial ulceration (Figure 1).

There was chalky white discharge from the ulceration. The patient had full, pain free range of motion of the elbow joint without any neurovascular deficit. The patient had multiple small swellings over the right pinna, bilateral hands, ankle and both feet. The systemic examination was unremarkable.

**Figure 2:**
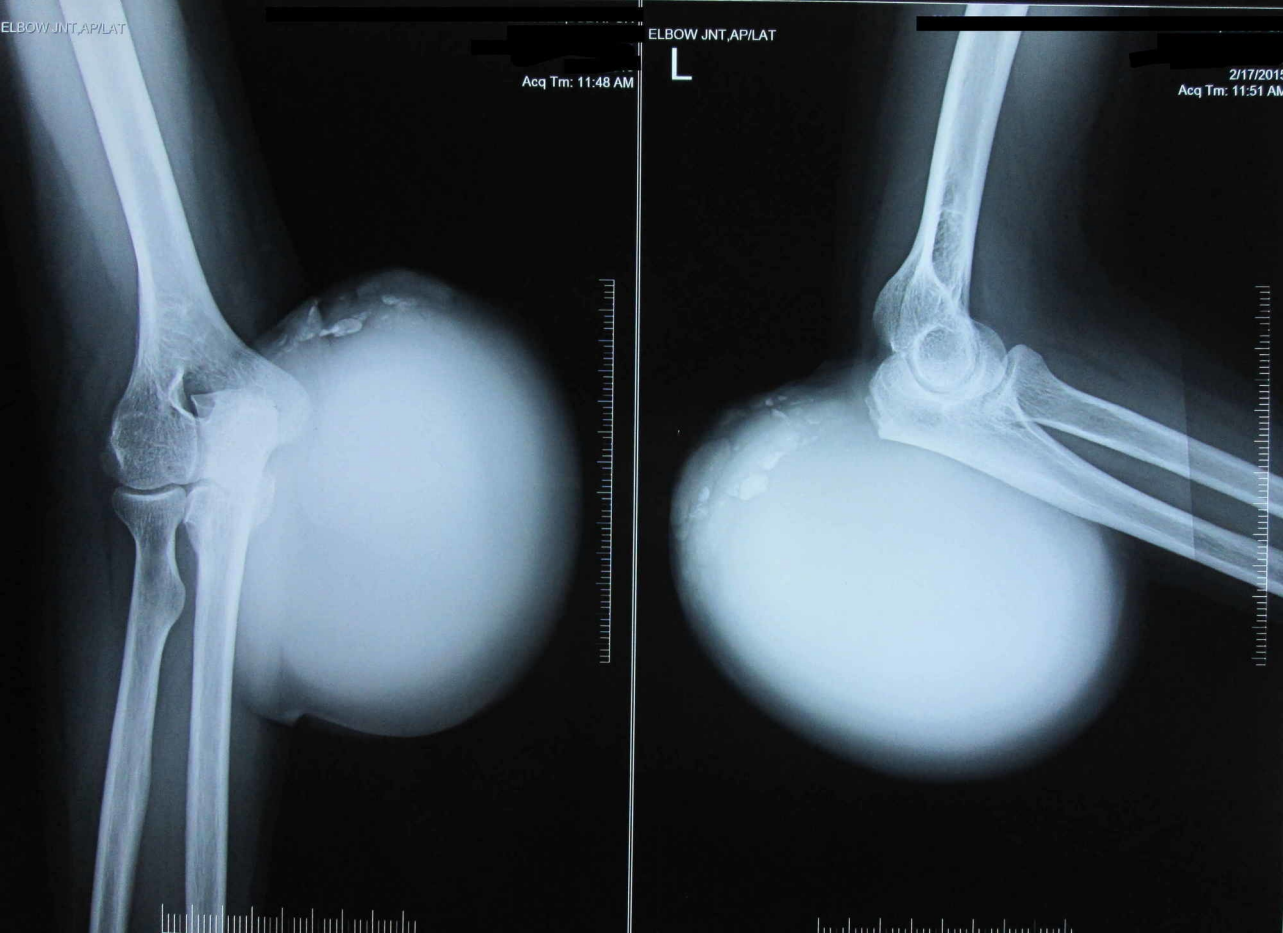
AP and lateral radiograph of the left elbow showing a huge soft tissue shadow with calcifications.

Hematological investigations revealed raised serum uric acid level (11 mg/dl), erythrocyte sedimentation rate of 38 mm/hour for the first hour and highly sensitive C-reactive protein level of 44.92 mg/l. Radiographs of the left elbow showed a huge soft tissue shadow with calcification (Figure 2).

**Figure 3:**
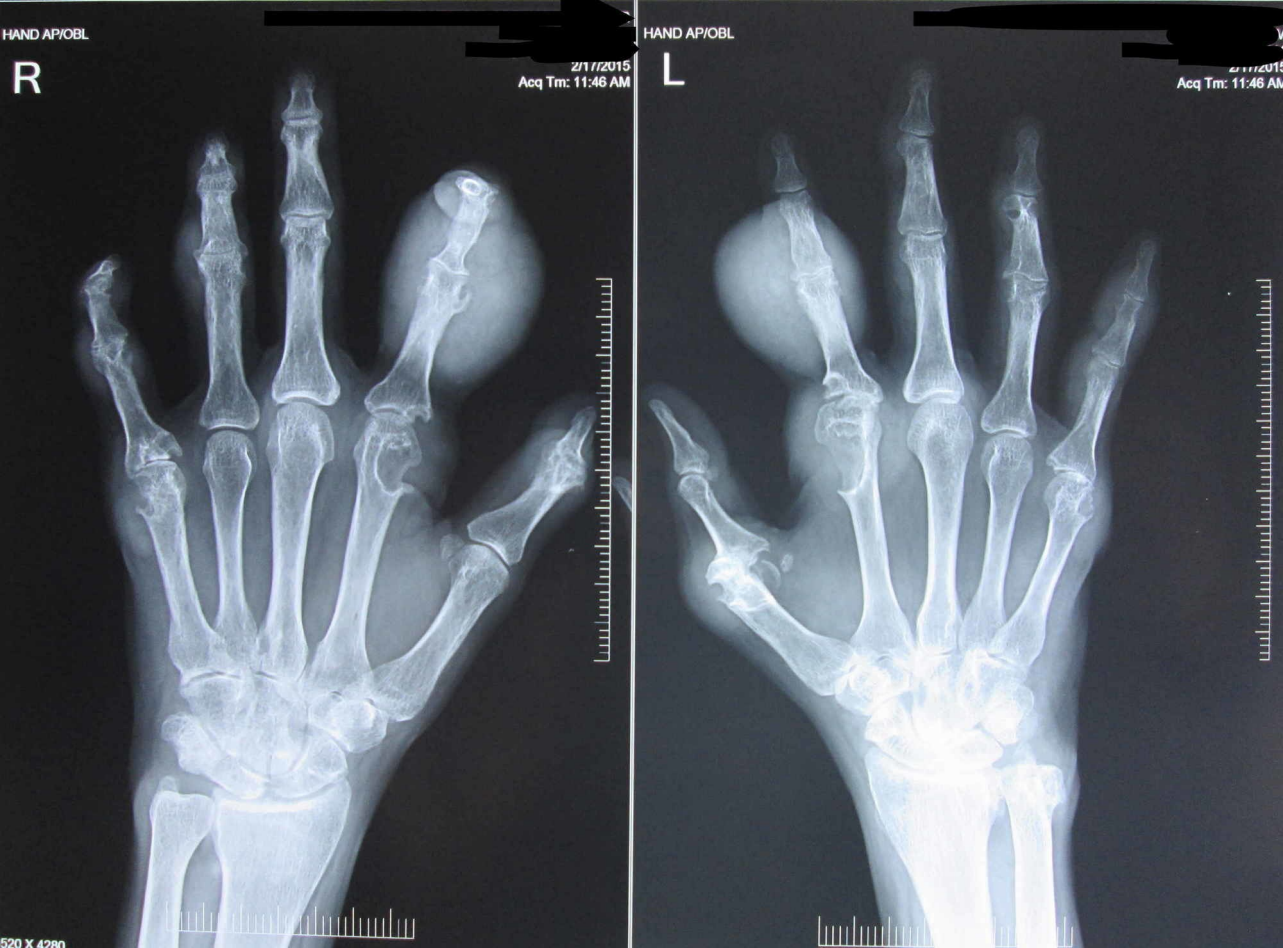
AP radiograph of bilateral hands showing soft tissue shadows in phalanges and periarticular punched-out erosions.

Radiographs of bilateral hands, ankle and feet showed similar soft tissue shadows in phalanges and periarticular punched-out erosions (Figure 3).

Direct fine needle aspiration cytology was done from the left elbow swelling, which yielded blood mixed brownish material, and from the right index finger swelling, which yielded whitish chalky material. On microscopic examination, smears from both the sites showed similar cytological material comprising of numerous scattered and aggregates of non-branching needle-shaped urate crystals in a fluffy to amorphous dirty background. Few scattered histiocytes and occasional lymphocytes were also visualized along with red blood cells.

The patient was initially put on dietary restrictions, plenty of fluids and drug therapy in the form of anti-inflammatory medications and oral allopurinol 100 mg three times daily for three months. At the end of three months, the serum uric acid levels reduced to 6.6 mg/dl, tophi over the pinna disappeared, the swellings over the hand and feet decreased in size, but the swelling and ulceration over the left elbow tophi continued to increase in size.

**Figure 4:**
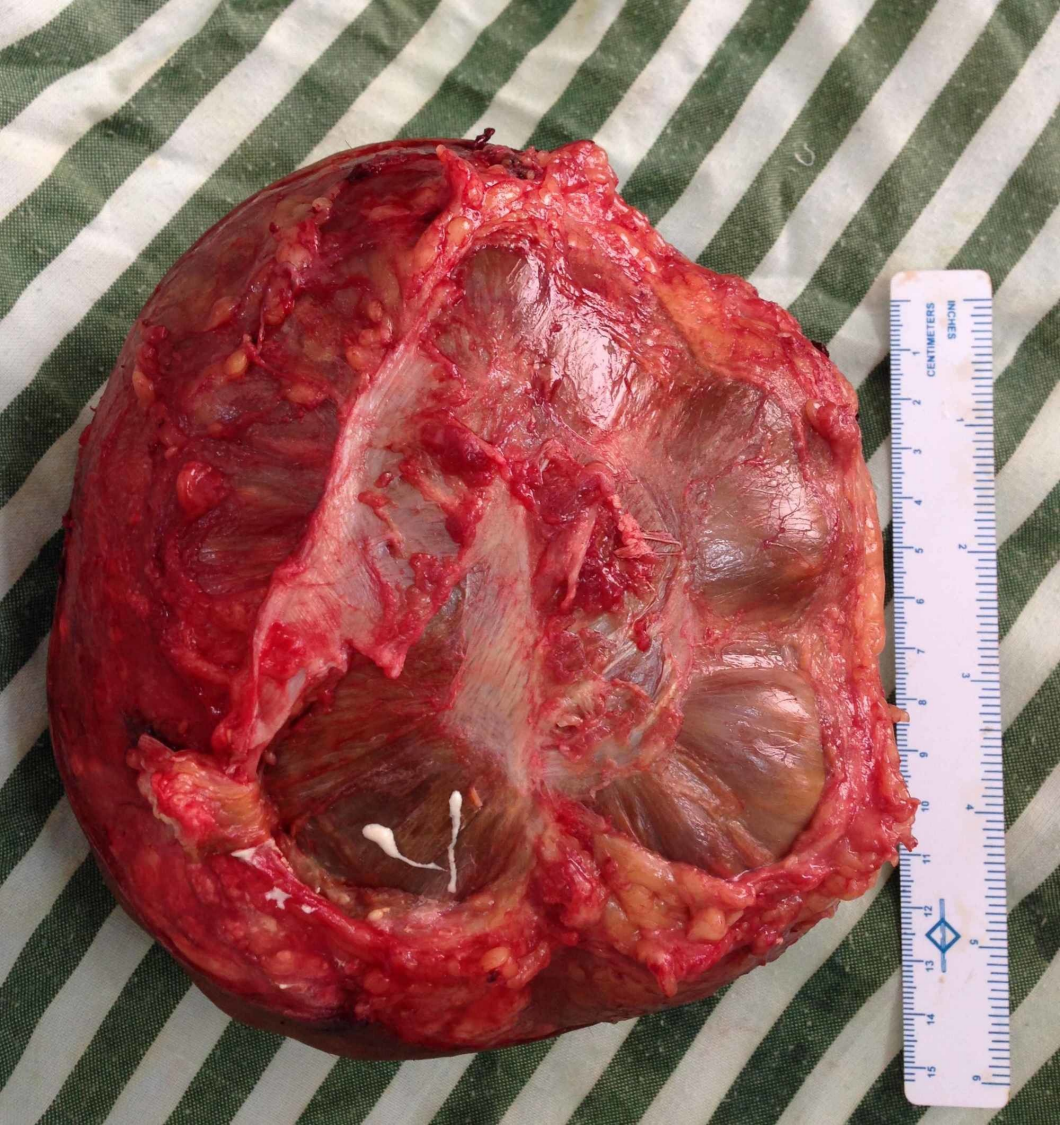
Excised mass weighing around 1,500 g.

After informed consent, the patient was planned for surgical excision of the massive left elbow tophi. The patient was positioned in a right lateral position under general anesthesia and en bloc excision of the swelling was performed through a standard posterior approach. The excised mass weighing around 1.5 kg was sent for histopathological examination and the wound was closed after excising the redundant skin margins (Figure 4).

**Figure 5:**
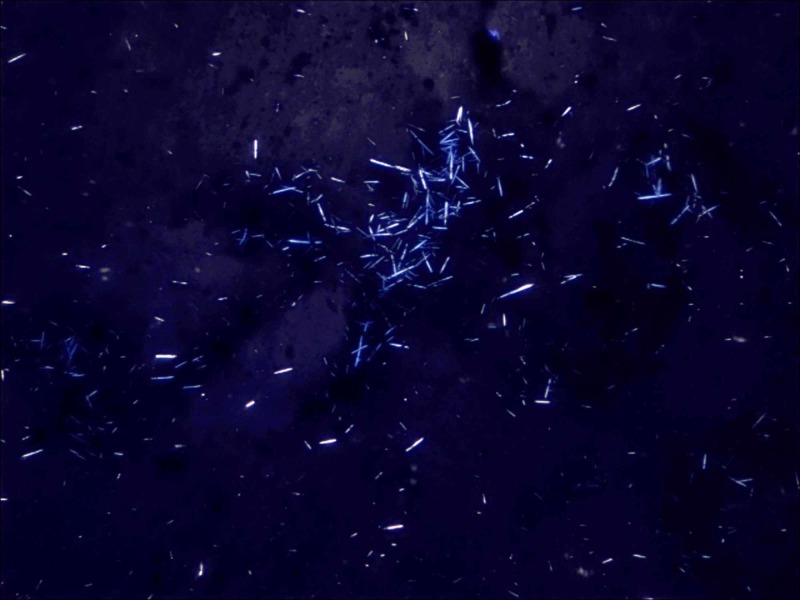
Imprint smear of the exudative material showing negatively birefringent needle-shaped sodium urate crystals.

Imprint smear of the exudative material from the specimen confirmed gouty tophi with negatively birefringent needle-shaped sodium urate crystals (Figure 5).

**Figure 6:**
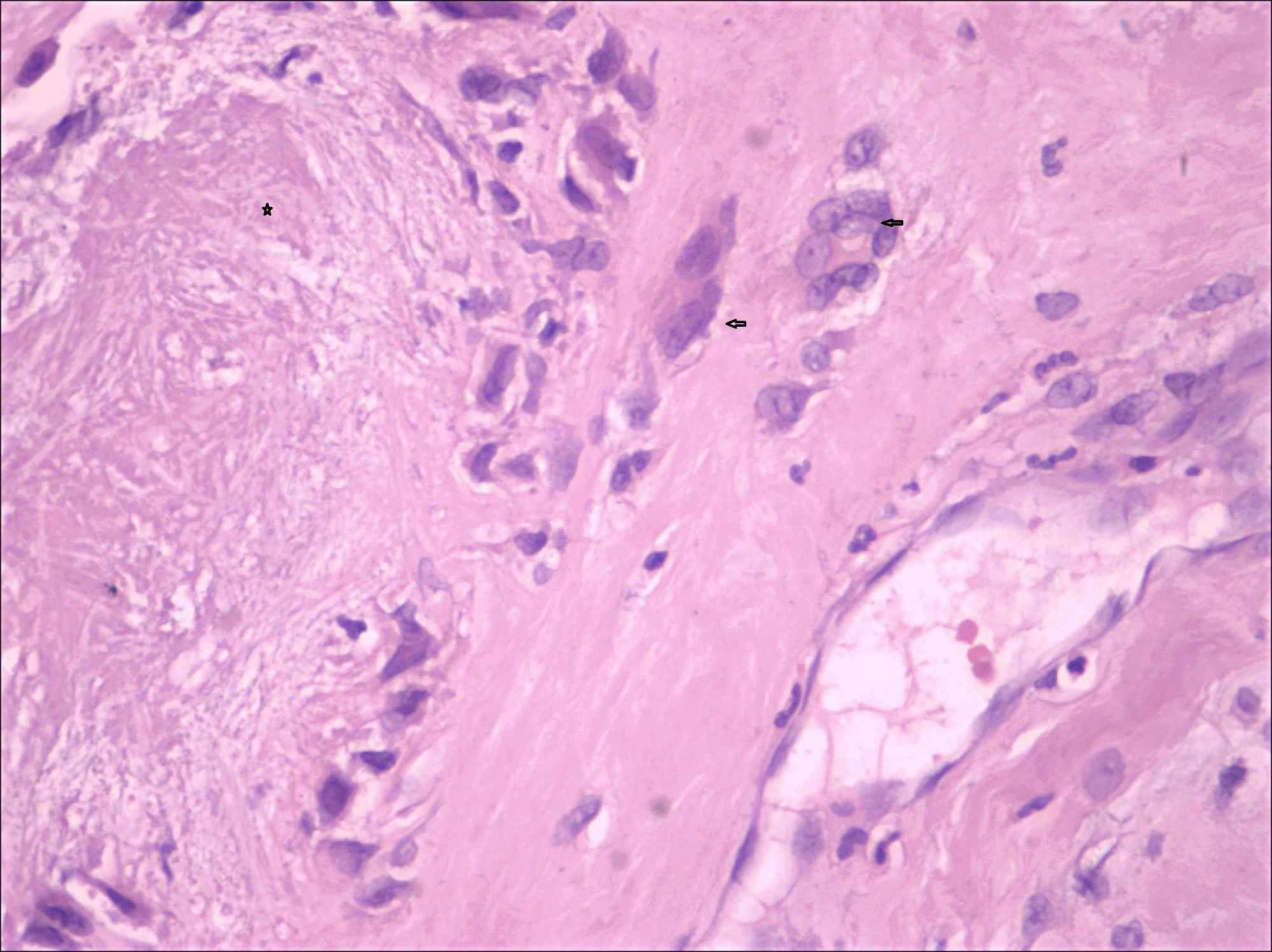
Histopathology image showing crystalline deposits (marked *) associated with multinucleated giant cells (marked arrow) and chronic inflammatory cells (H&E ×40).

Gross examination of soft tissue specimen revealed a skin-covered globular mass measuring 16x16x8 cm with an area of ulceration. On sectioning, a thick paste-like brownish material with whitish chalky deposits was observed. Microscopic examination showed skin with hyperkeratosis, parakeratosis and an ulcer covered by acute inflammatory exudate, crystalline deposits and fibrin and dense perivascular lymphocytic infiltrate in the dermis. Extensive crystal deposition (mainly needle-shaped crystals present in sheaves and bunches) and associated calcification within the dermis and fibrocollagenous areas were associated with multinucleated giant cell reaction and chronic inflammatory cells. These features were consistent with urate arthropathy (Figure 6).

**Figure 7:**
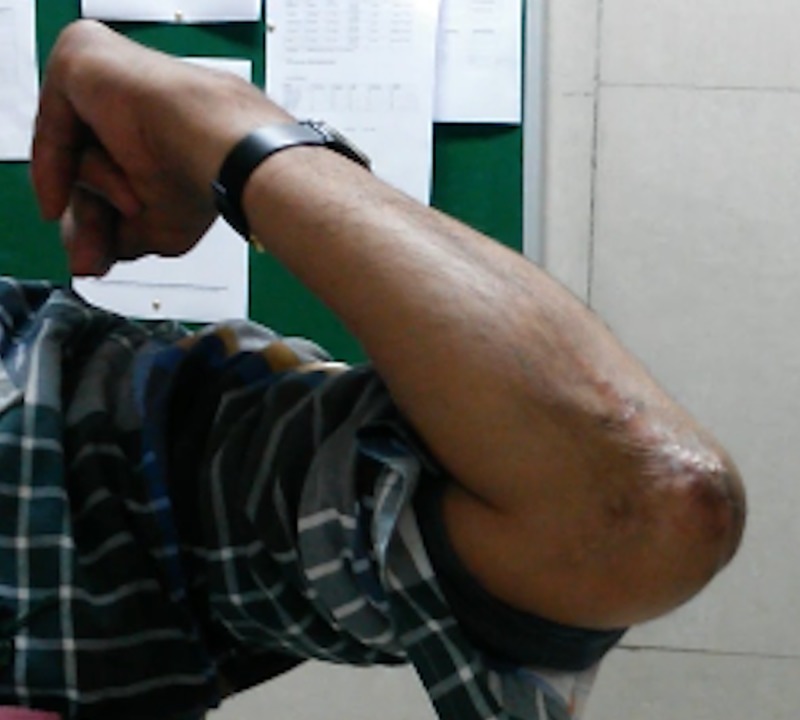
Clinical image showing healed scar and full function at the elbow.

Postoperatively, the wound healed uneventfully and at the last follow-up after two years, the patient had full range of motion without any neurovascular deficit (Figure 7).

## Discussion

Gout (also known as Podagra) is a systemic disorder of purine metabolism resulting in increased uric acid levels with recurrent attacks of arthritis [4,5]. The male-to-female ratio is 3.6:1 [6]. Chronic gouty arthritis is often associated with tophi, which are deposits of monosodium urate crystals in and around the joints and tendons. Distal extremities are most commonly involved with predilection for extensor surfaces. These tophi are considered pathognomonic for gout [7]. Conservative management with uricosuric drugs and xanthine oxidase inhibitors is effective in stabilizing and reducing the size of the tophi. However, 5%-10% of the patients do not respond completely to the conservative treatment [8].

The underlying pathology is invasion and destruction of skin, ligament, tendon, cartilage, and bone by deposition of urates. The process is accompanied by an acute or chronic inflammatory response at the site of involvement. The stages of gout include asymptomatic hyperuricemia, acute gouty arthritis, intercritical gout and chronic tophaceous gout which develops after 10 years of the intercritical gout phase [9,10].Tophi vary from semiliquid to inspissated, chalk-like deposits. These chalky materials reveal negatively birefringent needle-shaped crystals. 

Early diagnosis and treatment prevent severe crippling from the disease. Although majority of the cases respond to conservative management, relative surgical indications in chronic tophaceous gout are unsightly painful tophi, infection, impairment of tendon function, nerve compression due to tophi, impending skin necrosis, ulceration and discharging sinus, painful destruction of joint, a decrease in uric acid level in the body by excision of massive tophi and cosmesis [11-13]. Curettage and debridement can be done to remove tophi; however, it is associated with high rates of delayed wound healing and skin necrosis [14]. Other surgical methods include shaving, hydrotherapy and en bloc excision. We chose surgical treatment in our case as the mass was progressively increasing despite treatment, has developed ulceration and was cosmetically disfiguring. 

Postoperative wound healing is generally poor after excision of large tophi because of decreased circulation to the overlying skin which may require skin grafting [11]. In our case, the wound healed uneventfully as entire avascular skin was excised along with the mass. 

## Conclusions

This case highlights one of the rare presentations of chronic tophaceous gout with a massive elbow tophus weighing around 1.5 kg and locally mimicking a soft tissue tumor. A high index of suspicion is required in these cases, and a simple test like aspiration of the material for crystal analysis and cytology can help to distinguish this from soft tissue sarcoma. 
